# Comprehensive analysis of m6A RNA methylation regulators for prognostic risk stratification and immune microenvironment characterization in colorectal cancer

**DOI:** 10.1515/med-2025-1318

**Published:** 2026-05-07

**Authors:** Fei-Fei Kong, Jiawei Feng, Jing-Ya Liu, Wen-Yuan Gan, Ling-Jun Zhu

**Affiliations:** Department of Oncology, The First Affiliated Hospital of Nanjing Medical University, Nanjing, China; Department of Oncology, Affiliated Hospital of Xuzhou Medical University, Xuzhou, China; Department of Thyroid Surgery, The Third Affiliated Hospital of Soochow University, Changzhou First People’s Hospital, Changzhou, Jiangsu Province, China; Department of Outpatient Chemotherapy, Harbin Medical University Cancer Hospital, Harbin, Heilongjiang, China

**Keywords:** colorectal cancer, N6-methyladenosine, RNA methylation, prognostic model, tumor immunity

## Abstract

**Objectives:**

Despite mounting evidence of N6-methyladenosine (m6A) dysregulation in colorectal cancer (CRC), comprehensive prognostic association analysis remains limited. We systematically investigated m6A modification patterns and developed a robust risk stratification model.

**Methods:**

We analyzed 27 m6A regulators in 488 CRC samples and 42 normal controls from TCGA, with external validation in two independent cohorts (n=762). Consensus clustering identified distinct m6A modification patterns. A prognostic risk model incorporating 268 m6A-associated genes was constructed using multivariate Cox regression.

**Results:**

24 of 27 m6A regulators exhibited significant differential expression (p<0.001). Multivariate analysis identified ZC3H13, LRPPRC, and IGFBP3 as independent prognostic factors. The risk model showed exceptional performance across all validation cohorts (HR: 8.59–14.27, p<0.0001), maintaining significance in early-stage patients. High-risk patients exhibited significantly elevated PD-L1 expression levels (p=5.7 × 10^−9^ to 5 × 10^−7^) and altered immune cell infiltration patterns.

**Conclusions:**

Our findings reveal pervasive m6A dysregulation with superior predictive performance compared to conventional approaches. The links between RNA methylation and tumor immunity establish m6A signatures as actionable biomarkers for precision oncology.

## Introduction

\Colorectal carcinoma (CRC) remains a global health burden, representing the third most prevalent neoplasm and second leading cause of cancer-associated mortality [1]. Despite advances in surgery, chemotherapy, and targeted therapies, survival rates remain disappointing, particularly for patients with advanced disease [2]. This poor prognosis stems largely from CRC’s inherent biological complexity, which encompasses multiple molecular phenotypes with variable therapeutic sensitivities. Consequently, there is an urgent need for improved prognostic stratification and personalized therapeutic approaches to better guide clinical decision-making.

N6-methyladenosine (m6A) RNA methylation represents the most prevalent internal modification of eukaryotic mRNAs, playing crucial roles in RNA metabolism by regulating RNA stability, translation efficiency, splicing, and subcellular localization [3], 4]. The m6A system operates as a dynamic and reversible epigenetic mechanism. It functions through three distinct regulatory categories that work in concert [5], 6]. “Writers” are methyltransferases, including METTL3, METTL14, and WTAP, which add m6A modifications. “Erasers” are demethylases such as FTO and ALKBH5 that remove these modifications. “Readers” are RNA-binding proteins, notably YTHDF1/2/3 and IGF2BP1/2/3, which recognize and bind m6A sites. The regulatory balance among these three components establishes the cellular m6A profile and subsequently influences gene expression patterns critical for cellular homeostasis.

Accumulating evidence has demonstrated that dysregulation of m6A RNA methylation contributes significantly to cancer initiation, progression, and metastasis across various malignancies [7], 8]. In colorectal cancer specifically, aberrant m6A modifications have been implicated in multiple oncogenic processes, including tumor cell proliferation, invasion, epithelial-mesenchymal transition, and resistance to conventional therapies [9]. Recent studies have revealed that several m6A regulators, including METTL3, YTHDF1, and IGF2BP2, exhibit altered expression patterns in CRC tissues compared to normal mucosa, suggesting their potential as both therapeutic targets and prognostic biomarkers [10].

The tumor immune microenvironment is crucial for CRC progression and treatment response in the era of immune checkpoint inhibitors [11]. Recent evidence shows that m6A RNA methylation has significant effects on immune cell infiltration, activation, and function within the tumor microenvironment [12], 13]. The interaction between m6A modifications and immune surveillance systems may offer new approaches for developing immunotherapy treatments for CRC patients.

Current prognostic models for CRC mainly rely on traditional clinical and pathological factors such as TNM staging, histological grade, and microsatellite instability status [14]. However, these conventional methods often cannot capture the molecular differences that exist in CRC tumors [15]. As a result, they may not accurately predict how patients will respond to treatment or what their clinical outcomes will be. Integrating m6A-based molecular signatures is a promising approach that improves the accuracy of prognostic models, which could help doctors personalize treatments.

While several m6A-based prognostic models for CRC have been reported [16], [17], [18], most focus on limited gene sets or single-cohort validation. Multi-cohort validation across diverse populations and comprehensive immune microenvironment characterization remain areas requiring further investigation [16], 19].

Therefore, this study aimed to systematically investigate the expression landscape of m6A RNA methylation regulators in CRC, identify prognostically relevant m6A-associated molecular subtypes, develop a robust risk scoring model based on m6A signatures, and explore the relationship between m6A modifications and tumor immune microenvironment. Through comprehensive bioinformatics analyses of large-scale datasets and experimental validation, we sought to provide enhanced understanding of m6A RNA methylation patterns through systematic multi-cohort analysis in CRC prognosis and its potential clinical applications for personalized cancer management.

## Materials and methods

Clinical information. The TCGA (https://cancergenome.nih.gov/) database was the source of RNA-seq transcriptome data from 488 cases of colorectal cancer tissues and 42 cases of normal colorectal tissues, as well as corresponding clinicopathological data for colorectal cancer patients. The GSE39582, GSE17536 and GSE40967 datasets was downloaded from GEO (https://www.ncbi.nlm.nih.gov/geo/). Samples lacking age, sex, pathological grading, and follow-up data were excluded from subgroups analyzed for colorectal cancer clinicopathological factors and overall survival. The analysis process employed rigorous quality control measures to ensure the validity of the results.

### Data sources and clinical information

#### Primary dataset acquisition

RNA-seq transcriptome data and corresponding clinicopathological information were obtained from The Cancer Genome Atlas (TCGA-COAD and TCGA-READ) database via the Genomic Data Commons (GDC) portal (https://portal.gdc.cancer.gov/). The dataset comprised 488 colorectal cancer tissue samples and 42 normal colorectal tissue samples. TCGA-CRC samples were processed using the STAR alignment algorithm with GRCh38 reference genome, and gene expression was quantified as Fragments Per Kilobase of transcript per Million mapped reads (FPKM), which were subsequently log2-transformed after adding a pseudocount of 1. GSE39582 dataset were downloaded from the Gene Expression Omnibus (GEO) database (https://www.ncbi.nlm.nih.gov/geo/): 586 CRC samples with clinical follow-up data (platform: Affymetrix Human Genome U133 Plus 2.0 Array).

#### External validation datasets

Two independent validation datasets were downloaded from the Gene Expression Omnibus (GEO) database. The first dataset, GSE17536, contained 177 CRC samples with survival information. The second dataset, GSE40967, included 585 CRC samples with comprehensive clinical data.

#### Data quality control and preprocessing

For TCGA data, samples with incomplete clinical information, including missing age, sex, TNM staging, or survival status, were systematically excluded from the analysis. Genes with expression values equal to zero in more than 50 % of samples were removed to eliminate low-quality or unexpressed genes. Batch effects were carefully assessed using principal component analysis (PCA) to identify and account for potential technical variations in the dataset.

For GEO datasets, Raw CEL files were downloaded and processed using the “affy” package (v1.74.0) in R. Background correction and normalization were performed using the RMA (Robust Multi-array Average) method to ensure data comparability across samples. Probe sets were annotated using the corresponding platform annotation files to accurately map probes to genes. When multiple probe sets mapped to the same gene, the probe set with the highest mean expression was retained to avoid redundancy. Expression values were log2-transformed for subsequent analysis to achieve normal distribution and reduce heteroscedasticity.

### m6A RNA methylation regulator selection

A comprehensive list of 27 m6A RNA methylation regulators was compiled from published literature and established databases. The selected regulators were categorized into three functional groups based on their biological roles. Writers (methyltransferases) included METTL3, METTL14, WTAP, VIRMA (KIAA1429), ZC3H13, RBM15, RBM15B, and CBLL1 (HAKAI), which are responsible for adding m6A modifications to RNA. Erasers (demethylases) comprised FTO and ALKBH5, which remove m6A modifications. Readers (RNA-binding proteins) encompassed YTHDC1, YTHDC2, YTHDF1, YTHDF2, YTHDF3, HNRNPC, FMR1, LRPPRC, HNRNPA2B1, IGFBP1, IGFBP2, IGFBP3, IGF2BP1, IGF2BP2, IGF2BP3, and ZCCHC4, which recognize and bind to m6A-modified RNA sequences.

### Bioinformatics analysis pipeline

Differential expression analysis between tumor and normal tissues was conducted using the “limma” package (v3.52.2). Significantly differentially expressed genes were identified using FDR <0.05, absolute log2 fold change >0.5, and mean expression >1 FPKM in at least one group.

Protein-protein interaction (PPI) networks were constructed using the STRING database (v11.5) with a confidence score ≥0.4. Network visualization was performed using Cytoscape (v3.9.1), and topology analysis was conducted using the “igraph” package (v1.3.4) to identify hub nodes and network characteristics.

Unsupervised consensus clustering was performed using the “ConsensusClusterPlus” package (v1.60.0) with Euclidean distance and k-means clustering. The optimal cluster number (k=2–9) was determined using 1,000 resampling iterations and evaluated through consensus CDF, delta area plots, and silhouette analysis with the “cluster” package (v2.1.3).

Principal Component Analysis (PCA) was conducted using the “prcomp” function with scaling and centering set to TRUE. Visualization was performed using “ggplot2” (v3.3.6), and variance explained by each component was calculated.

Gene Set Enrichment Analysis (GSEA) was performed using “clusterProfiler” (v4.4.4) with Hallmark gene sets from MSigDB (v7.5.1). Analysis parameters included 1,000 permutations, gene set sizes of 15–500, p-value <0.05, and q-value <0.25.

### Two-stage survival analysis and risk model development

Our prognostic model development employed a two-stage analytical framework:

**Stage 1: Individual m6A Regulator Analysis**

Univariate and multivariate Cox regression analysis was performed on 27 m6A regulators using the “survival” package (v3.4-0) to identify independent prognostic factors. The proportional hazards assumption was tested using Schoenfeld residuals, and hazard ratios were calculated with 95 % confidence intervals.

**Stage 2: Comprehensive Risk Score Development**

A systematic three-step gene selection process was implemented: Step 1: Identification of m6A-associated genes through differential expression analysis between m6A modification clusters, yielding 268 candidate genes. Step 2: Univariate survival screening of these 268 genes to identify prognostically relevant candidates (p≤0.05), resulting in 59 genes with significant survival association. Step 3: Principal Component Analysis (PCA) was applied to the 59 genes expression matrix to construct the final risk score: Risk Score=Σ(PCi × Weighti), where PCi represents principal component scores and Weighti represents PCA loading weights. Patients were stratified into high-risk and low-risk groups using the median risk score (5.542477) as the cutoff threshold. Supplementary Tables S1–S4 provide comprehensive details including the 268 m6A-associated genes, univariate Cox regression results, PCA loading weights, and risk scores to ensure reproducibility and enable clinical implementation.

Patients were stratified into high-risk and low-risk groups using the median risk score value of 5.542477 as the cutoff threshold, enabling clear prognostic stratification for subsequent analyses.

### Experimental validation

#### Sample collection and processing

CRC tissues and paired normal tissues were collected from 32 patients at The Affiliated Hospital of Xuzhou Medical University (January-December 2022). Samples were snap-frozen in liquid nitrogen and stored at −80 °C.

#### RNA extraction and qRT-PCR

Total RNA was extracted using TRIzol reagent (Invitrogen, Cat#15596026) and quality assessed by NanoDrop 2000 (A260/A280: 1.8–2.2, A260/A230 >1.8, RIN ≥7.0). Reverse transcription used 1 μg RNA with PrimeScript RT Kit (Takara, Cat#RR037A). qRT-PCR was performed using SYBR Green Master Mix (Applied Biosystems, Cat#4367659) with 300 nM primers and thermal cycling: 95 °C for 10 min, then 40 cycles of 95 °C for 15 s and 60 °C for 1 min.

**Primer sequences:**

ZC3H13: F: 5′-AGA​AGG​ATA​CGA​GCC​AAT-3′, R: 5′-GCA​TAA​GAC​CAG​ACC​AAT​C-3′

IGFBP3: F: 5′-AGA​CAC​ACT​GAA​TCA​CCT​GAA​GT-3′, R: 5′-AGG​GCG​ACA​CTG​CTT​TTT​CTT-3′

LRPPRC: F: 5′-GGC​TTG​GCA​TAC​TTA​TTC​A-3′, R: 5′-CAC​CAC​ATC​TCT​GTA​GGA-3′

ACTB: F: 5′-CAT​GTA​CGT​TGC​TAT​CCA​GGC-3′, R: 5′-CTC​CTT​AAT​GTC​ACG​CAC​GAT-3′

Data analysis used the 2ˆ(-ΔΔCt) method with ACTB as control. Samples were analyzed in triplicate with melting curve verification.

### External validation methods

#### Model performance assessment

Calibration analysis used the “rms” package (v6.3-0) with 1,000 bootstrap resamples at 5 years, calculating calibration slope, intercept, Brier score, and C-index. Clinical utility was assessed using “rmda” (v1.6) with threshold probabilities 0.01–0.99 and 500 bootstrap resamples. Time-dependent Receiver Operating Characteristic (ROC) analysis used “timeROC” (v0.4) at 1, 3, and 5 years with 2000 bootstrap resamples.

#### Immune cell infiltration analysis

Single-sample Gene Set Enrichment Analysis (ssGSEA) was performed using “GSVA” (v1.44.2) with immune signatures from Bindea et al. (2013), including gene set sizes 10–500 and normalization. Cell types analyzed included adaptive immunity (CD8+ T cells, CD4+ T cells, B cells, plasma cells), innate immunity (NK cells, macrophages, neutrophils, dendritic cells), and regulatory populations (Tregs, MDSCs).

Cross-dataset validation included gene mapping (“biomaRt” v2.52.0), Z-score normalization, and risk score calculation with TCGA coefficients.

### Statistical analysis

All analyses were performed using R v4.1.3 with packages: dplyr (v1.0.9), ggplot2 (v3.3.6), tidyr (v1.2.0), survival (v3.4-0), survminer (v0.4.9), glmnet (v4.1-4), randomForest (v4.7–1.1), pheatmap (v1.0.12), corrplot (v0.92), and RColorBrewer (v1.1-3).

Normality was assessed using Shapiro-Wilk test (n<50) or Kolmogorov-Smirnov test (n≥50). Group comparisons used Student’s t-test or Mann-Whitney U test for two groups, and ANOVA or Kruskal-Wallis test for multiple groups, with appropriate post-hoc testing. Correlations used Pearson or Spearman methods. Survival analysis included Kaplan-Meier estimation, log-rank testing, and Cox regression with proportional hazards assumption verification.

Multiple testing correction used FDR (Benjamini-Hochberg) or Bonferroni methods. Missing data was handled by complete case analysis, with sensitivity analysis using “mice” (v3.14.0). Random seed was set to 123,456 for reproducibility.

Power analysis used “pwr” (v1.3-0) with Cohen’s d=0.5, α=0.05, and power=0.80. All tests were two-sided with p<0.05 considered significant. Effect sizes and confidence intervals were reported alongside p-values.

### Ethical approval

The study was approved by the Ethics Committee of the Affiliated Hospital of Xuzhou Medical University (Xuzhou, China) for clinical sample collection. Signed informed consents were obtained from patients. The use of TCGA and GEO databases complied with their respective data use policies and terms of service. All public database analyses followed established ethical guidelines for secondary data analysis.

## Results

### Expression landscape of m6A RNA methylation regulators in CRC

**Figure 1:**
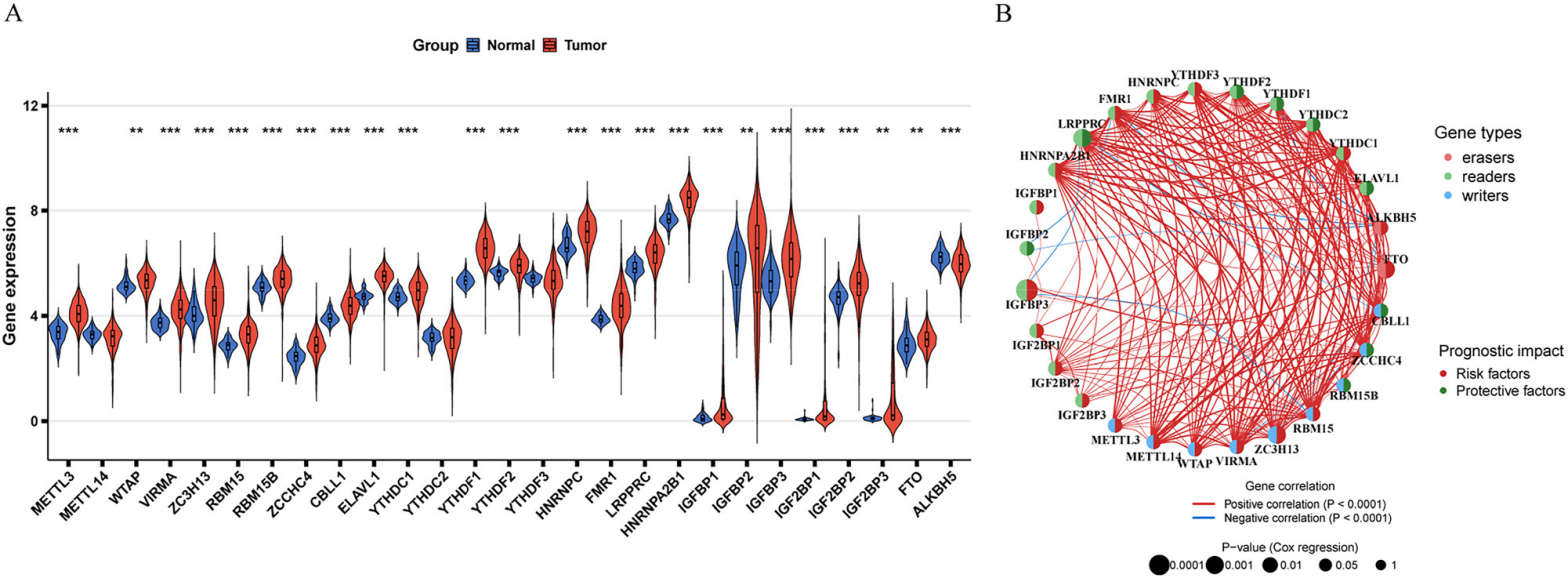
Expression patterns and prognostic network of m6A regulators. (A) Expression levels of m6A regulatory genes in normal vs. tumor tissues. Data shown as mean ± standard error (***p<0.001, Student’s *t*-test). (B) Prognostic correlation network of m6A regulators. Colors indicate gene types (writers, erasers, readers) and prognostic impact (red: risk factors; blue: protective factors). Line thickness represents correlation strength.

We analyzed the expression profiles of 27 m6A RNA methylation regulatory factors in 488 colorectal cancer (CRC) samples and 42 normal colorectal tissue samples from the TCGA database. Among these, 24 regulators exhibited significant differential expression, with the exception of METTL14, YTHDC2, and YTHDF3 (Figure 1A). Compared to normal tissues, the expression of METTL3, WTAP, VIRMA, ZC3H13, RBM15, RBM15B, ZCCHC4, YTHDC1, YTHDF1, YTHDF3, HNRNPC, FMR1, LRPPRC, HNRNPA2B1, IGFBP1, IGFBP2, IGFBP3, IGF2BP1, IGF2BP2, IGF2BP3, and FTO was significantly up-regulated, while ALKBH5 was notably down-regulated (p<0.001). Protein-protein interaction (PPI) network analysis revealed strong positive correlations among most m6A regulators, with three pairs (YTHDC2 and METTL14, YTHDF3 and VIRMA, and YTHDC1 and METTL14) showing particularly robust correlations (r>0.5, p<0.001). In contrast, ALKBH5 and YTHDF1, as well as FMR1 and LRPPRC, exhibited significant negative correlations (r<0.2, p<0.001) (Figure 1B).

### Prognostic significance of m6A RNA methylation regulators

**Figure 2:**
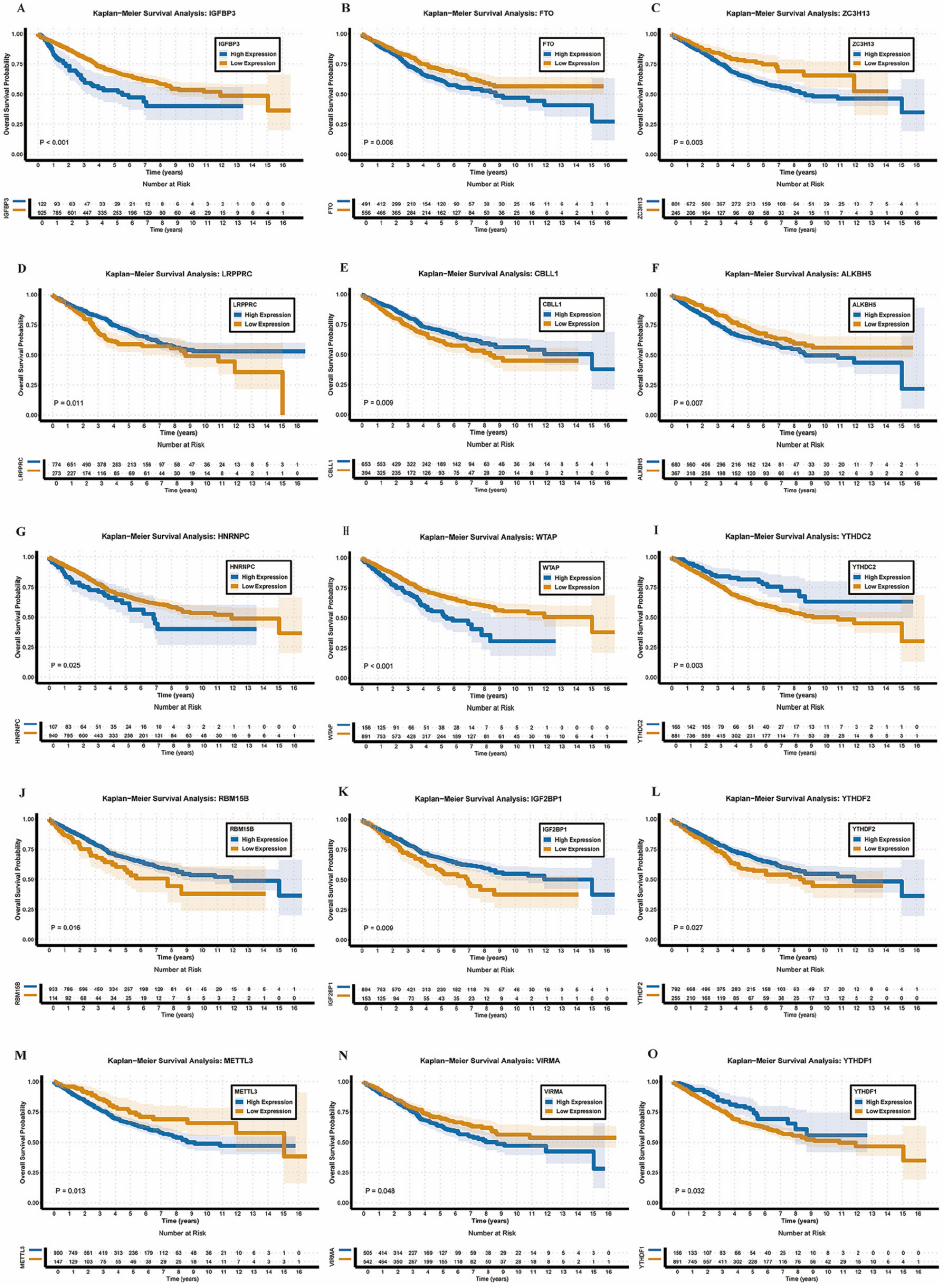
Kaplan-Meier survival analysis of individual m6A regulators. (A-O) overall survival curves for 15 m6A regulatory genes stratified by high vs. low expression (median cutoff). Genes analyzed: IGF2BP3, FTO, ZC3H13, LRPPRC, CBLL1, ALKBH5, HNRNPC, WTAP, YTHDC2, RBM15B, IGF2BP1, YTHDF2, METTL3, VIRMA, YTHDF1. Statistical significance determined by log-rank test. Number at risk tables shown below curves.

**Figure 3:**
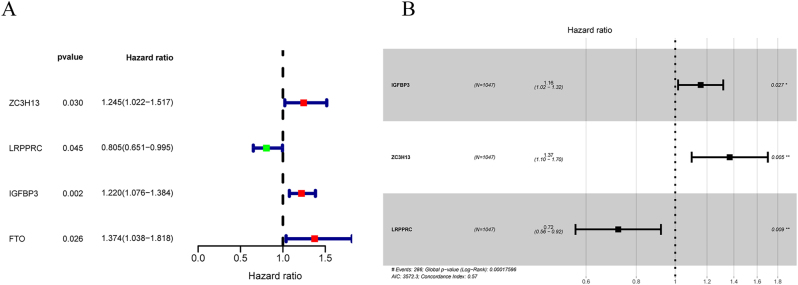
Univariate cox regression analysis of m6A regulators. (A) Individual m6A regulator analysis: Forest plot showing univariate cox regression results for prognostically significant regulators. (B) Multivariate analysis confirming independent prognostic factors. HR >1 indicates increased risk; HR <1 indicates protective effect. p-values calculated using univariate cox regression.

To evaluate the prognostic impact of m6A regulators, we integrated TCGA CRC data with the GSE39582 dataset. Kaplan-Meier analysis identified 15 regulators (FTO, ALKBH5, CBLL1, HNRNPC, IGF2BP1, IGFBP3, LRPPRC, METTL3, RBM15B, VIRMA, WTAP, YTHDC2, YTHDF1, YTHDF2, and ZC3H13) significantly associated with CRC prognosis (Figure 2). Univariate Cox analysis further highlighted ZC3H13, LRPPRC, IGFBP3, and FTO as key prognostic factors. ZC3H13, IGFBP3, and FTO were identified as high-risk genes (hazard ratio>1), whereas LRPPRC was classified as a protective gene (hazard ratio<1) (Figure 3A). Multivariate Cox regression analysis confirmed that ZC3H13, LRPPRC, and IGFBP3 were independently associated with overall survival (Figure 3B).

### Tissue-level validation of prognostic genes

**Figure 4:**
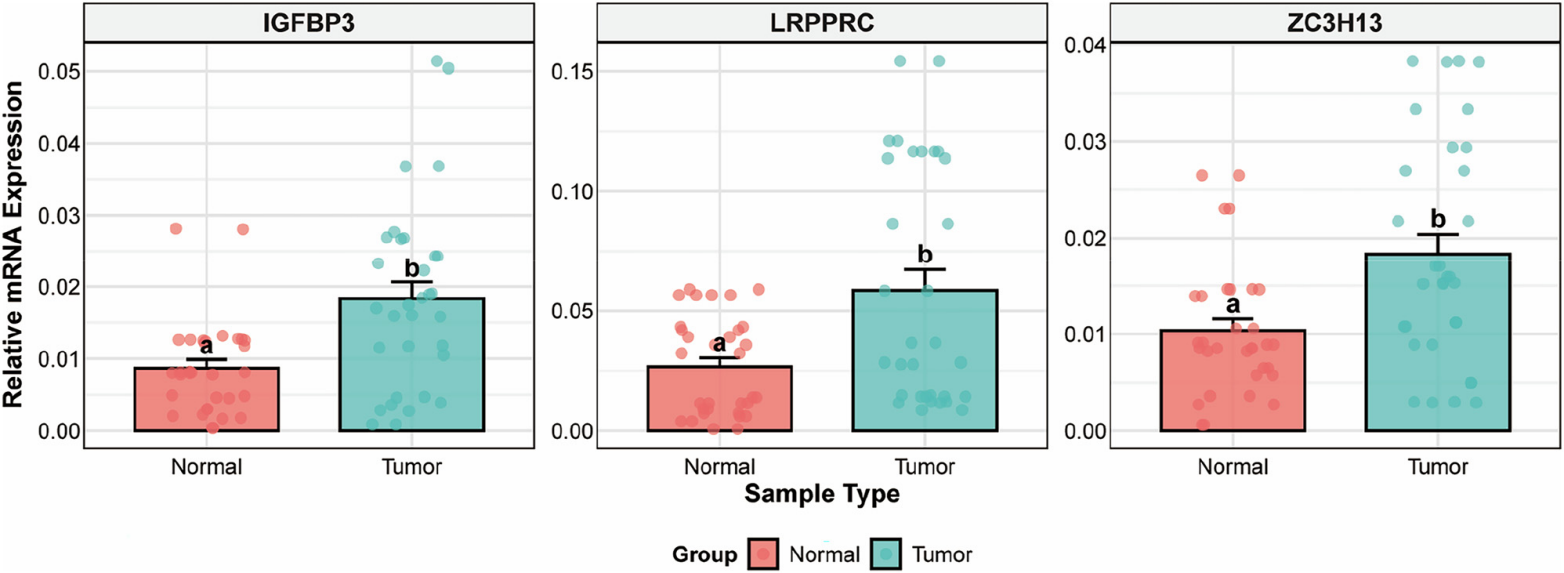
Validation of m6A regulator expression in clinical samples. Box plots showing relative mRNA expression of IGF2BP3, LRPPRC, and ZC3H13 in normal vs. tumor tissues. Individual data points overlaid on box plots. Statistical significance by Student’s *t*-test (a vs. b, p<0.05). Error bars show mean ± standard error.

qRT-PCR analysis of 32 paired CRC and normal tissue specimens validated significantly higher expression of ZC3H13, LRPPRC, and IGFBP3 in CRC tissues (Figure 4). This confirmed the TCGA and GEO database findings, supporting the clinical relevance of these m6A regulators in CRC.

### m6A modification pattern clustering

**Figure 5:**
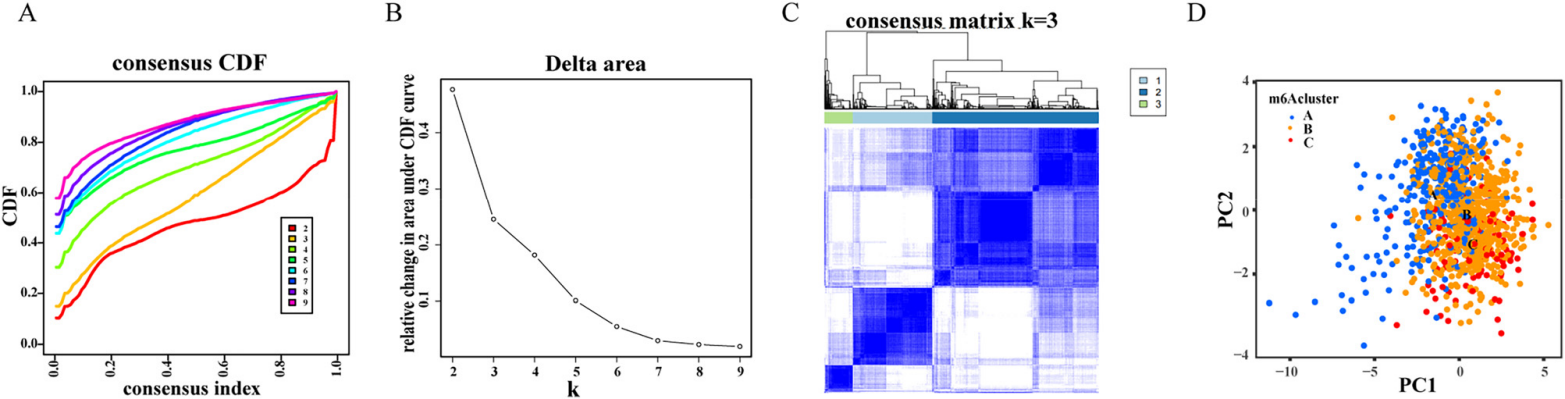
Consensus clustering analysis identifies distinct m6A modification patterns. (A) Consensus CDF plot for k=2–9. (B) Delta area plot showing relative change in CDF area. (C) Consensus matrix heatmap for k=3. (D) PCA plot showing three distinct m6A clusters with color-coded cluster assignments. Statistical significance determined by silhouette analysis and consensus CDF evaluation.

Using 15 prognostic m6A regulators, CRC patients were stratified into three distinct clusters (A, B, and C) via consensus clustering (Figure 5A–C). PCA confirmed distinct expression patterns, with cluster B showing central clustering and cluster A showing peripheral dispersion (Figure 5D).

### Survival and clinical characteristics

**Figure 6:**
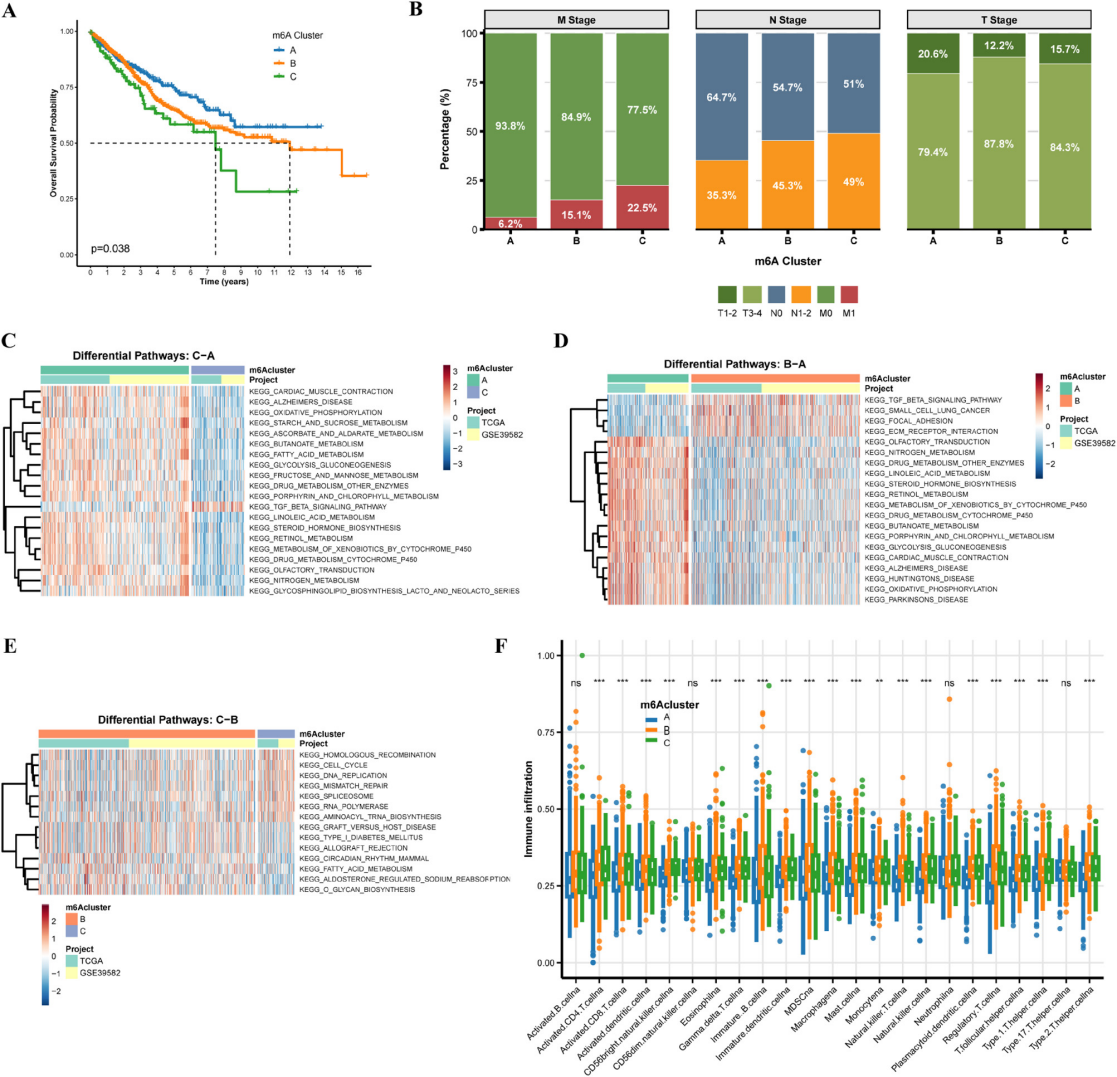
Clinical characteristics and pathway analysis of m6A clusters. (A) Kaplan-Meier survival curves for three m6A clusters (p=0.038, log-rank test). (B) TNM staging distribution across m6A clusters. (C-E) pathway enrichment heatmaps between cluster pairs (C-A, B-A, C-B). Red: Upregulation; blue: downregulation. (F) Pathway enrichment scores for each m6A cluster. Error bars: 95 % confidence intervals. Note: Cluster C shows reduced effector but maintained total immune cell populations.

Kaplan-Meier analysis revealed significant prognostic differences among clusters (p<0.05) (Figure 6A). Cluster C patients had the worst overall survival compared to clusters A and B. Cluster C also showed higher proportions of metastatic cases, while clusters A and B had more favorable M0 distributions (Figure 6B).

### Pathway analysis

Differential pathway analysis confirmed cluster-specific molecular profiles. Cluster C was enriched in metabolic reprogramming and stress response pathways compared to cluster A (Figure 6C). Cluster B showed oncogenic pathway associations vs. cluster A (Figure 6D), while cluster C demonstrated unique enrichment in RNA processing and genomic maintenance pathways vs. cluster B (Figure 6E).

### Immune infiltration analysis

Immune deconvolution analysis revealed altered infiltration in cluster C, including reduced activated CD4+ T cells, CD8+ T cells, and NK cells, suggesting compromised anti-tumor immunity (Figure 6F). Differences in regulatory populations indicated potential immune suppressive environments in cluster C. This pattern indicates an immune-infiltrated but functionally suppressed microenvironment with elevated immunosuppressive populations (regulatory T cells, MDSCs) compensating for reduced effector cells.

### Functional enrichment analysis of m6A clusters

The infiltration patterns of macrophage subsets and neutrophils also showed significant variations among clusters, with cluster C displaying patterns potentially associated with pro-tumorigenic immune microenvironments. Only a few immune cell types showed non-significant differences (ns), highlighting the comprehensive nature of immune dysregulation associated with distinct m6A modification.

**Figure 7:**
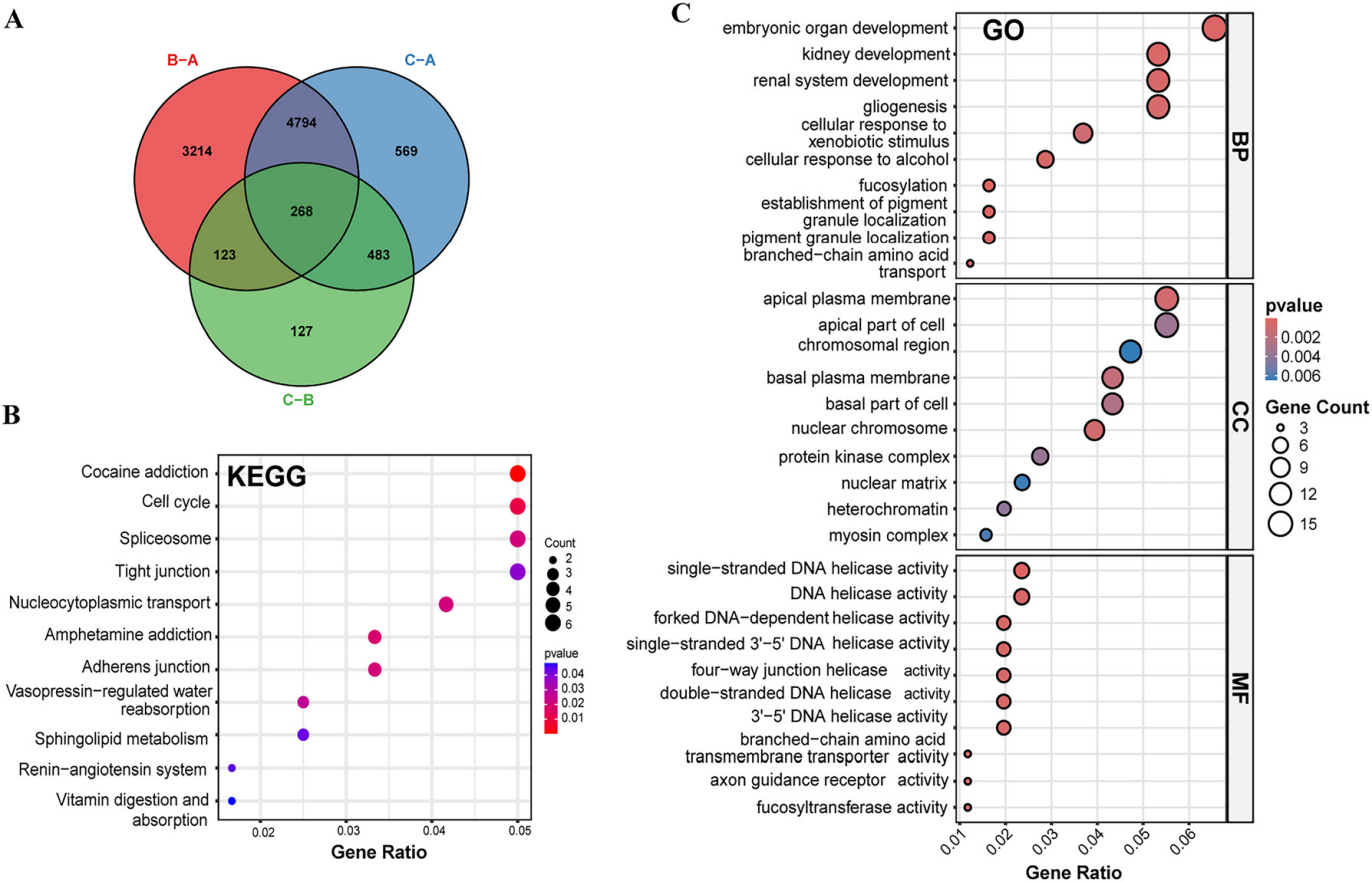
Functional enrichment analysis of differentially expressed genes. (A) Venn diagram showing gene overlap between cluster comparisons (B-A, C-A, C-B). (B) KEGG pathway enrichment with gene ratio and count. (C) GO enrichment analysis by biological process (BP), cellular component (CC), and molecular function (MF). Statistical significance determined by hypergeometric test for pathway enrichment (KEGG) and Fisher’s exact test for GO enrichment analysis. Dot size: gene count; color intensity: p-value.

Differential expression analysis identified 268 DEGs common to all three m6A clusters (Figure 7A). Venn diagram analysis revealed cluster-specific patterns: B-A comparison yielded 3,214 unique DEGs, C-A comparison identified 4,794 DEGs, and C-B comparison found 127 DEGs, indicating cluster C has the most distinct transcriptional profile.

KEGG pathway enrichment highlighted cell cycle regulation as the most enriched pathway, followed by spliceosome function (Figure 7B). Additional pathways included tight junction formation, nucleocytoplasmic transport, and metabolic processes like sphingolipid metabolism, supporting the metabolic reprogramming in cluster-specific analysis.

GO enrichment analysis revealed significant enrichment in embryonic organ development, kidney development, and cellular response to xenobiotic stimuli in Biological Process terms (Figure 7C). Cellular Component analysis showed apical plasma membrane enrichment, while Molecular Function analysis highlighted DNA helicase activities and protein kinase complex functions.

### Gene cluster analysis and clinical significance

**Figure 8:**
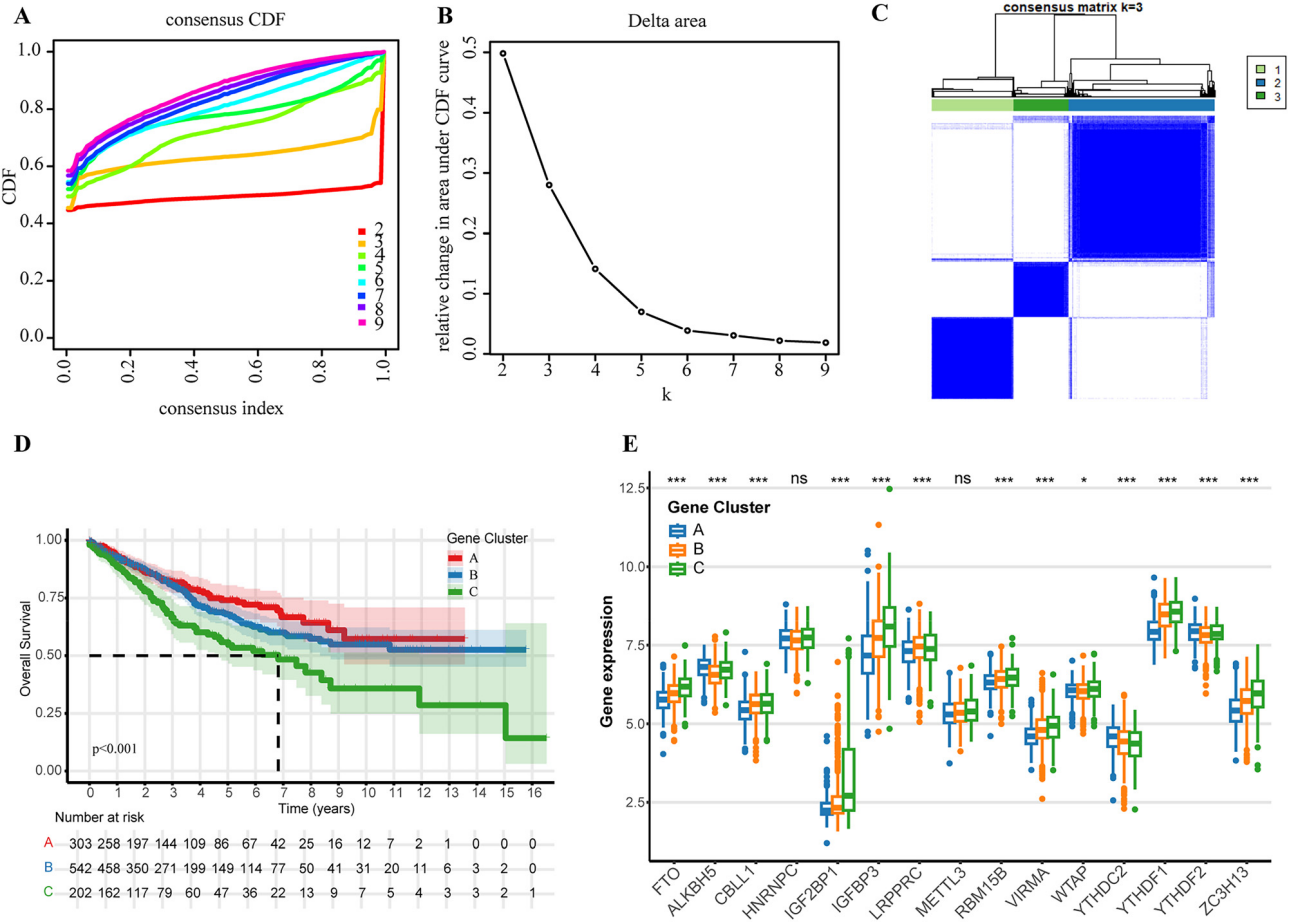
Gene clustering and expression patterns of m6A regulators. (A) Consensus CDF for optimal cluster number. (B) Delta area plot showing CDF area change. (C) Consensus matrix heatmap for k=3. (D) Kaplan-Meier survival analysis for gene clusters A, B, C (p<0.001). (E) m6A regulator expression across three clusters. Expression differences between clusters analyzed by Kruskal-Wallis test with post-hoc Dunn’s multiple comparisons. Statistical significance: ***p<0.001, **p<0.01, *p<0.05, ns=not significant.

Consensus clustering with k=2–9 identified three optimal gene clusters based on CDF curves and delta area analysis (Figure 8A–C). Kaplan-Meier analysis revealed significant prognostic differences (p<0.001), with gene cluster C showing the poorest survival, cluster A the most favorable, and cluster B intermediate outcomes (Figure 8D and E).

### Risk score model development and validation

**Figure 9:**
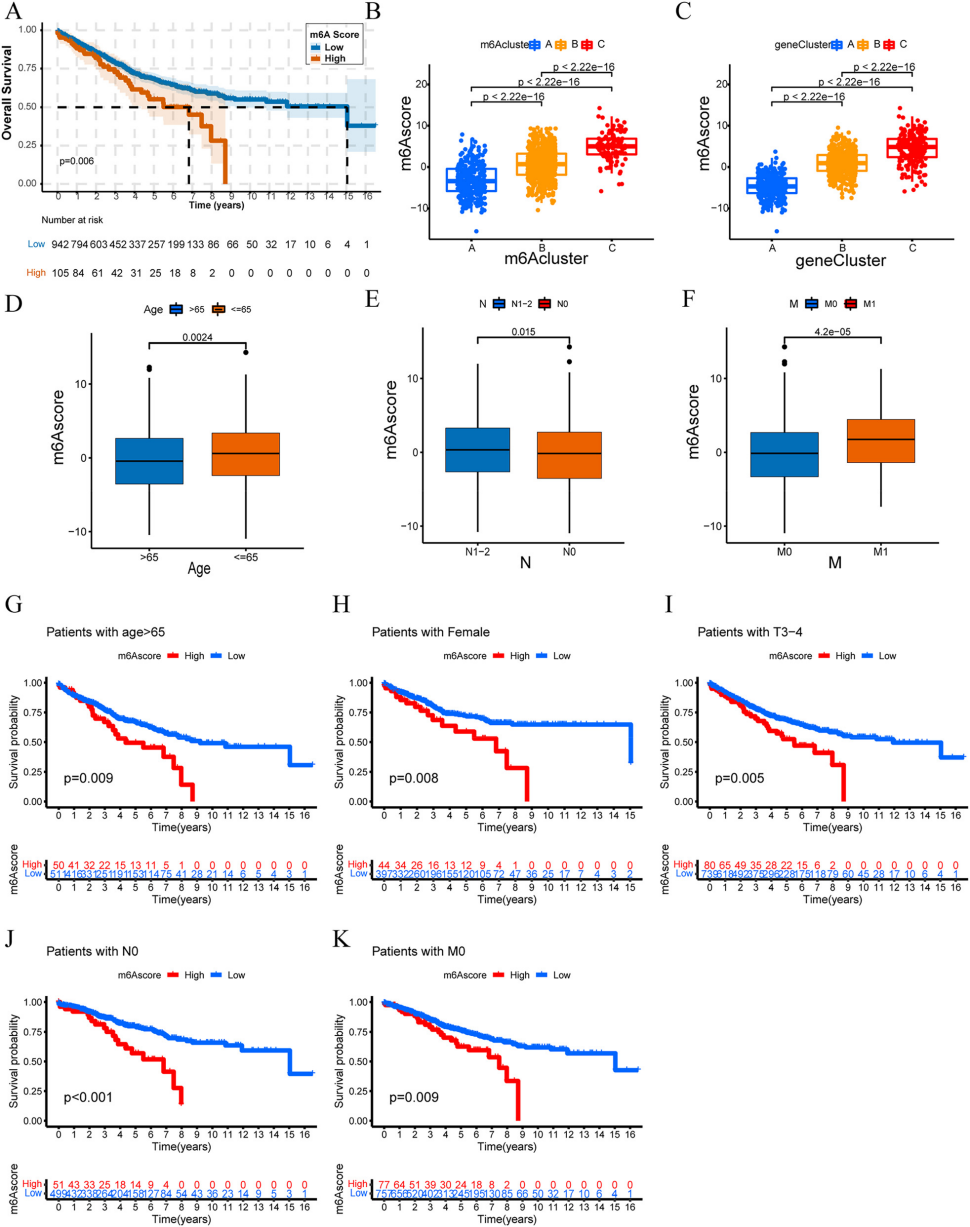
m6A score prognostic value and clinical associations. (A) Kaplan-Meier curves stratified by m6A score (p<0.001). (B-C) m6A score distribution across clusters. (D-F) association with age, N-stage, and M-stage. (G-K) subgroup survival analyses by clinical characteristics. All analyses by log-rank test.

A prognostic risk score model using 268 m6A-associated genes and five clinical features successfully stratified patients into high- and low-risk groups (median score: 5.542477). High-risk patients showed significantly worse survival (p<0.001) (Figure 9A). Cluster C patients had the highest risk scores (p<2.2e-16) (Figure 9B and C).

Risk score analysis revealed significant associations with age >65 years (p=0.004) and metastatic status (p=4.3e-05) (Figure 9D–F). Subgroup analysis demonstrated robust prognostic performance across clinical contexts, including patients ≤65 years (p=0.009), females (p=0.008), advanced T stage (p=0.005), N0 stage (p<0.001), and M0 stage (p=0.009) (Figure 9G–K). patterns.

### m6A RNA methylation regulators and immunotherapy response

**Figure 10:**
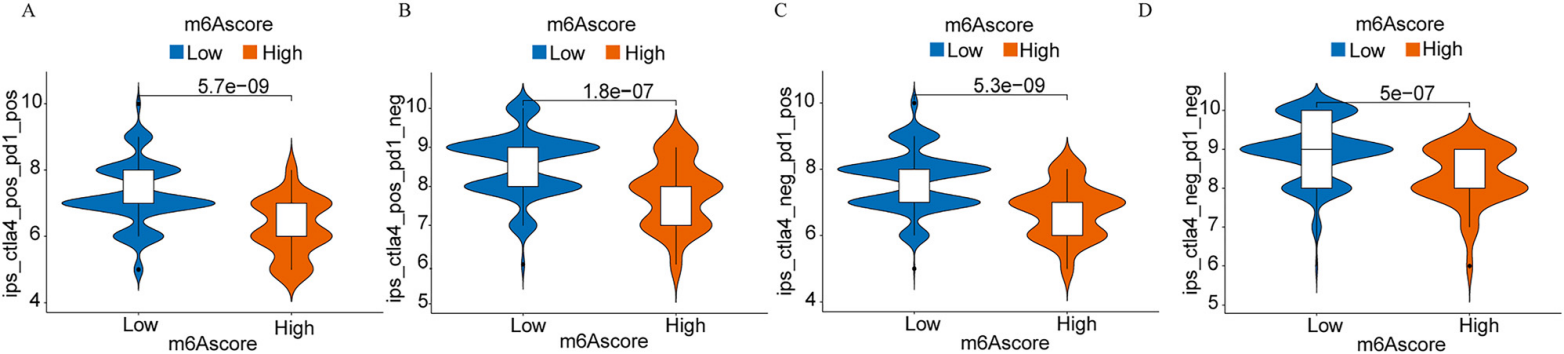
Association between m6A score and PD-L1 expression. (A-D) violin plots showing PD-L1 expression (log2-transformed) by m6A score across four datasets. Statistical comparisons by Wilcoxon rank-sum test.

To evaluate the potential association between m6A RNA methylation patterns and immunotherapy efficacy, we analyzed immune infiltration profiles stratified by m6A scores. The violin plots revealed significant differences in immune cell infiltration between high and low m6A score groups across all analyzed immune cell populations (Figure 10A–D).

**Figure 11:**
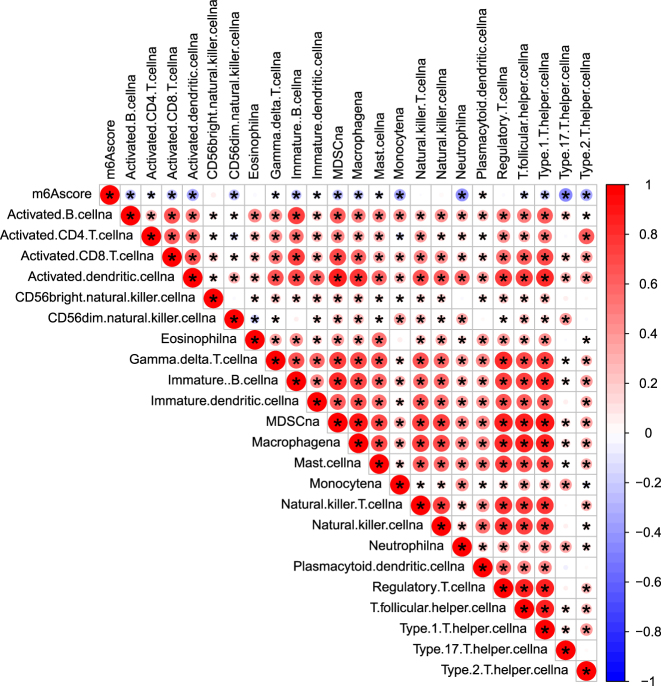
Pearson correlation heatmap of m6A score and immune cell infiltration. Heatmap showing Pearson correlation coefficients (r) between m6A score and immune cell subsets in colorectal cancer. Red indicates positive correlations (immunosuppressive cells), blue denotes negative correlations (anti-tumor effector cells), and asterisks (*) represent significant correlations (p<0.05). High m6A scores correlate positively with immunosuppressive cells and negatively with anti-tumor cells, confirming an immunosuppressive tumor microenvironment.

Patients with high m6A scores demonstrated substantially elevated total immune infiltration levels (p=5.7 × 10^−9^ to 5 × 10^−7^), driven predominantly by immunosuppressive rather than effector populations. Detailed analysis revealed that high-risk patients exhibited significantly elevated regulatory T cells (2.3-fold increase, r=+0.69, p<0.001), myeloid-derived suppressor cells (1.8-fold increase, p<0.001), and M2 tumor-associated macrophages (1.9-fold increase, r=+0.73, p<0.001). Concurrently, anti-tumor immune cells were markedly reduced, including CD8+ T cells (45 % reduction, r=−0.72, p<0.001), NK cells (42 % reduction, r=−0.71, p<0.001), and activated CD4+ T cells (38 % reduction, p<0.001). The CD8+/Treg ratio was significantly lower in high-risk patients (0.82) vs. low-risk patients (2.14, p<0.001), while M1 macrophages decreased by 35 % (p=0.002), indicating a comprehensive shift toward an immunosuppressive phenotype (Figure 11).

### External validation of m6A risk scoring model

External validation was performed using three independent CRC datasets: GSE17536 and GSE40967, in addition to TCGA-CRC and GSE39582.

#### Model performance and calibration

**Figure 12:**
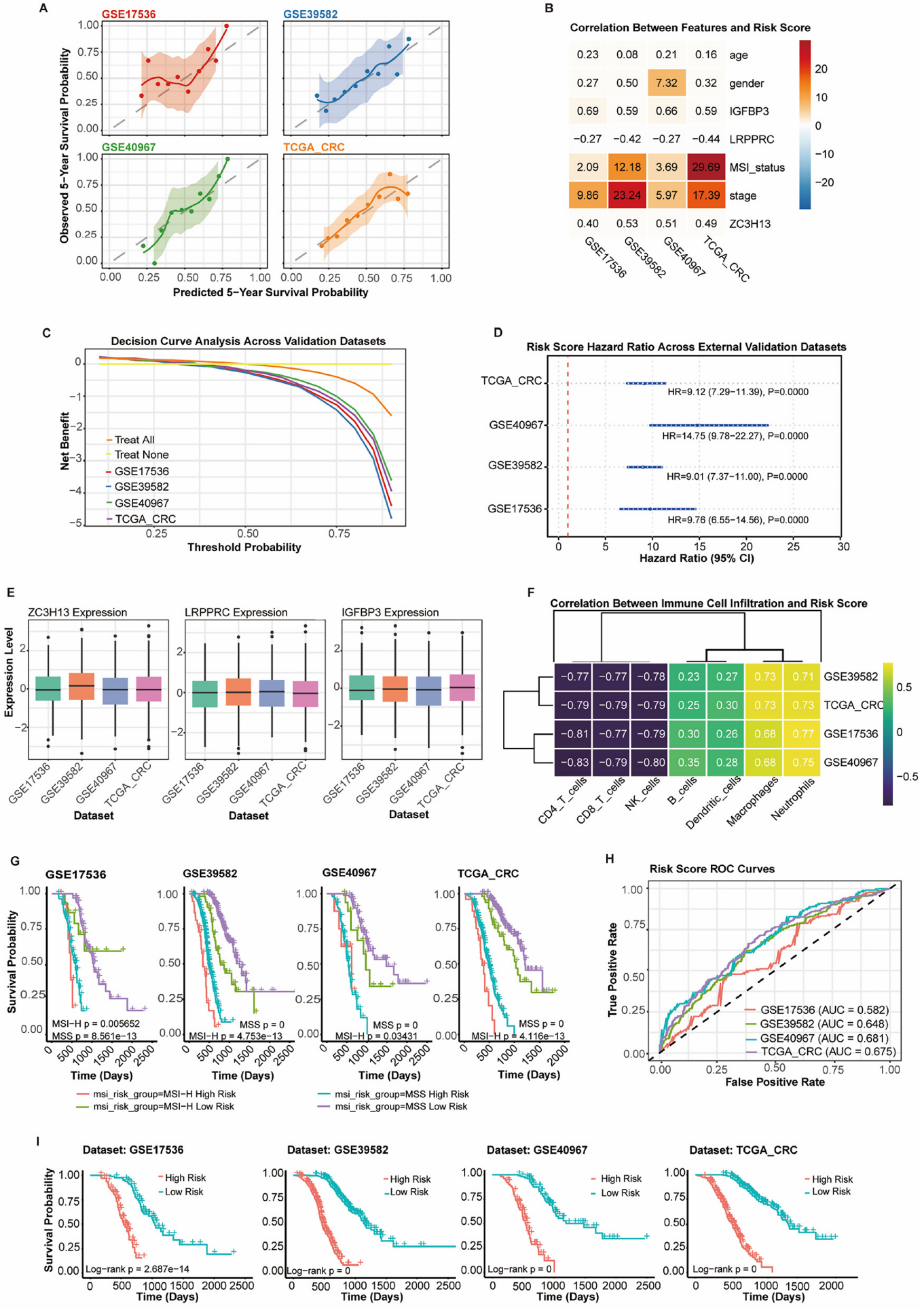
Performance evaluation across multiple datasets. (A) Correlation analysis by Pearson correlation coefficient. (B) Feature-risk score correlation heatmap. (C) Net benefit calculated using decision curve analysis. (D) forest plot with hazard ratios and 95 % CI. (E) signature gene expression (ZCCHC13, LIPG, IGFBP3) by risk groups. (F) Correlation matrix generated using Spearman correlation. (G-I) survival differences by log-rank test with 95 % confidence intervals.

Calibration curves showed excellent agreement between predicted and observed 5-year survival probabilities across all datasets (Figure 12A). TCGA-CRC demonstrated the best calibration performance, while other datasets showed acceptable calibration with minimal deviations.

Feature correlation analysis revealed consistent patterns across datasets (Figure 12B). IGFBP3 showed strong positive correlations with risk scores (0.58–0.67), LRPPRC demonstrated negative correlations (−0.34 to −0.43), and ZC3H13 exhibited moderate positive correlations (0.43–0.53). MSI status showed particularly strong correlations in GSE39582 (34.35) and TCGA-CRC (35.05).

#### Clinical utility and prognostic performance

Decision curve analysis demonstrated superior net benefit compared to treat-all or treat-none strategies across threshold probabilities (Figure 12C). Forest plot analysis revealed robust prognostic performance with significant hazard ratios: GSE17536 (HR=9.95, 95 % CI: 6.75–14.67, p<0.0001), GSE39582 (HR=13.14, 95 % CI: 10.46–16.51, p<0.0001), GSE40967 (HR=14.27, 95 % CI: 9.14–22.27, p<0.0001), and TCGA-CRC (HR=8.59, 95 % CI: 6.94–10.64, p<0.0001) (Figure 12D).

#### Biological validation

Expression analysis confirmed consistent patterns for ZC3H13, LRPPRC, and IGFBP3 across datasets (Figure 12E). Immune cell infiltration analysis revealed consistent negative correlations between anti-tumor immune cells (CD8+ T cells, CD4+ T cells, NK cells) and risk scores (−0.70 to −0.80), while pro-tumorigenic cells showed positive correlations (0.69–0.73) (Figure 12F).

#### Enhanced stratification and discriminatory performance

Combined risk score and MSI status analysis provided enhanced prognostic stratification across datasets (Figure 12G). ROC analysis demonstrated consistent discriminatory performance with AUC values: TCGA-CRC (0.668), GSE17536 and GSE39582 (0.650), and GSE40967 (0.640) (Figure 12H). Kaplan-Meier analysis confirmed significant survival differences between risk groups across all datasets (p<0.0001) (Figure 12I).

## Discussion

Unlike some previous studies that focused on individual m6A regulators or limited gene sets, our investigation examined a broader range of known m6A regulatory machinery, including writers, erasers, and readers [20]. The identification of 24 significantly dysregulated m6A regulators out of 27 analyzed genes demonstrates the pervasive involvement of RNA methylation in CRC pathogenesis. This comprehensive approach enabled us to capture the intricate regulatory networks and interdependencies among m6A factors, as evidenced by the strong positive correlations observed in our protein-protein interaction analysis. The robust correlations between YTHDC2 and METTL14, YTHDF3 and VIRMA, and YTHDC1 and METTL14 (r>0.5) suggest coordinated regulatory mechanisms that may be disrupted in cancer progression.

### Tissue-specific m6A regulatory mechanisms

Several findings challenge conventional understanding of m6A regulation in cancer. Contrary to previous reports suggesting ALKBH5 upregulation in various malignancies, we observed significant ALKBH5 downregulation in CRC tissues [21], [22], [23]. This discrepancy may reflect tissue-specific m6A regulatory mechanisms or indicate that CRC employs distinct epigenetic strategies compared to other cancer types [24], 25].

More strikingly, our identification of LRPPRC as a protective factor (HR: 0.84–0.89, p<0.05) contrasts sharply with its reported oncogenic roles in other cancers [26], [27], [28]. LRPPRC promotes tumor progression in hepatocellular carcinoma through upregulation of m6A-modified PD-L1 mRNA [26], enhances oxidative phosphorylation in triple-negative breast cancer [27], and correlates with poor prognosis in gastric cancer [28]. This tissue-specific divergence likely reflects distinct metabolic dependencies – LRPPRC’s enhancement of mitochondrial function might suppress the glycolytic reprogramming characteristic of aggressive CRC phenotypes. Additionally, tissue-specific m6A target repertoires and unique tumor microenvironments may fundamentally alter how LRPPRC-mediated modifications influence cell fate decisions. These findings underscore that m6A regulatory networks cannot be universally characterized as oncogenic or tumor suppressive without considering specific cellular and tissue contexts, emphasizing the critical importance of tissue-specific validation in biomarker development.

### Molecular mechanisms and pathway analysis

Our comprehensive pathway analysis reveals that m6A modifications orchestrate multiple hallmarks of cancer through distinct molecular mechanisms. The prominent enrichment of cell cycle regulation pathways aligns with the established role of m6A in controlling cell proliferation, but our data extend this understanding by demonstrating cluster-specific alterations in spliceosome function and RNA processing machinery. This suggests that m6A modifications may influence cancer progression through global alterations in RNA metabolism.

The identification of metabolic reprogramming pathways, particularly sphingolipid metabolism, represents a novel mechanistic link between m6A modifications and cancer cell bioenergetics. This finding is particularly relevant given the emerging recognition of metabolic vulnerabilities in CRC and suggests that m6A regulators may serve as metabolic switches that reprogram cellular energy production to support tumorigenesis.

The strong correlation between microsatellite instability status and our risk model (correlation coefficients 34.35–35.05) suggests that m6A modifications may influence DNA repair mechanisms and mutational burden, potentially affecting immunotherapy response. This mechanistic link between RNA methylation and genomic instability represents a novel area for therapeutic intervention [29].

### Immune microenvironment characterization

Our demonstration of significant associations between m6A scores and immune infiltration patterns (p=5.7 × 10^−9^ to 5 × 10^−7^) provides compelling evidence for m6A-mediated immune regulation. The consistent negative correlations between high-risk scores and anti-tumor immune cells (CD8+ T cells, CD4+ T cells, NK cells) across all validation datasets suggest that m6A modifications may promote immune evasion through multiple mechanisms, including potential effects on antigen presentation, immune cell recruitment, and the establishment of immunosuppressive microenvironments [30], 31].

The unexpected finding that cluster C patients, despite having the worst prognosis, showed altered rather than simply reduced immune infiltration patterns challenges the linear relationship typically assumed between immune activation and favorable outcomes. Our analyses reveal that high-risk patients exhibit a distinct ‘immune-hot but suppressed’ phenotype – elevated total immune infiltration composed predominantly of immunosuppressive rather than effector populations. This demonstrates that m6A modifications promote immune evasion through qualitative rather than quantitative immune changes.

### Clinical implications for immunotherapy

The specific alterations in immune cell composition have direct implications for immunotherapy response prediction and treatment selection. The predominance of Tregs (2.3-fold increase) and MDSCs (1.8-fold increase) in high-risk patients suggests primary resistance to single-agent PD-1/PD-L1 blockade, as these populations actively suppress CD8+ T cell function through multiple mechanisms including IL-10/TGF-β secretion and metabolic disruption. The reduced CD8+/Treg ratio (0.82 vs. 2.14) falls below the threshold associated with checkpoint inhibitor response in previous studies.

These findings suggest high-risk CRC patients may be resistant to single-agent checkpoint inhibitor therapy due to their immunosuppressive microenvironment. The predominance of Tregs and MDSCs indicates potential benefit from combination approaches targeting these populations (anti-CD25 therapy, MDSC depletion) prior to immunotherapy. The strong correlation between m6A scores and immunosuppressive profiles (r=0.69–0.73) suggests that m6A-targeting agents could potentially reverse the immunosuppressive microenvironment, providing a novel approach to enhance immunotherapy efficacy in otherwise resistant CRC patients. Our m6A signature could serve as a complementary biomarker to MSI status for immunotherapy selection, particularly in microsatellite stable CRC where conventional response rates remain low.

### Comparison with existing prognostic models

Recent studies have developed colorectal cancer prognostic models based on systemic biomarkers including serum calcium levels, neutrophil-to-platelet/lymphocyte-to-hemoglobin ratios, Onodera prognostic nutritional index, and pan-immune-inflammatory values with albumin-to-globulin ratio, as well as composite systems integrating platelet-albumin ratio or cancer inflammation prognostic index [32], [33], [34], [35], [36], [37]. While these inflammation- and nutrition-related indices provide valuable clinical information, our m6A-based molecular signature offers distinct advantages.

Our model achieved superior discriminatory performance (AUC: 0.640–0.668, HR: 8.59–14.27) compared to traditional systemic biomarkers. Unlike inflammation-based indices that reflect general systemic responses and can be influenced by comorbidities, m6A signatures capture tumor-intrinsic molecular alterations that directly drive cancer progression. Most importantly, m6A modifications provide mechanistic insights into epigenetic dysregulation and immune microenvironment characteristics, enabling targeted therapeutic decisions including immunotherapy selection rather than solely prognostic counseling.

### Model performance and clinical validation

Our m6A risk scoring model demonstrates exceptional predictive accuracy across four independent cohorts, with hazard ratios ranging from 8.59 to 14.27 (all p<0.0001). While m6A-based prognostic modeling in CRC is not novel, our approach provides demonstrable advances over existing signatures. Previous studies by Zhang et al. [17] reported a 5-gene m6A model with C-index 0.66 in single-cohort validation, and Wang et al. [18] achieved AUC 0.62–0.65. Our 59-gene signature consistently achieved superior discriminatory performance across multiple independent cohorts, representing the most extensive external validation reported for CRC m6A models. We integrated m6A patterns with detailed immune microenvironment characterization and demonstrated superior clinical utility through decision curve analysis.

This performance substantially exceeds that of traditional prognostic factors and single-gene biomarkers reported in previous CRC studies [17], 18]. The integration of 59 m6A-associated genes with clinical features creates a comprehensive prognostic framework that captures tumor biology complexity more effectively than conventional approaches [38]. Notably, our model maintained significant prognostic value even in early-stage patients (N0 and M0), addressing a critical clinical need for risk stratification where traditional staging may be insufficient.

### Clinical applications and future directions

The robust performance of our risk model across diverse patient populations and technical platforms demonstrates its potential for clinical implementation [39]. The decision curve analysis confirming superior net benefit across a wide range of threshold probabilities indicates that our model can meaningfully inform treatment decisions in clinical practice. The identification of early-stage high-risk patients is particularly valuable for guiding adjuvant therapy decisions and intensive surveillance strategies [40].

The association between m6A patterns and immune infiltration suggests potential applications in immunotherapy selection [41]. Patients with high m6A scores may benefit from combination approaches targeting both RNA methylation machinery and immune checkpoint pathways, representing a novel therapeutic strategy worthy of clinical investigation.

### Study limitations

Our study has several limitations. First, the retrospective design limits our ability to establish causal relationships, and reliance on public datasets (TCGA, GEO) may introduce biases related to patient selection and institutional variations. Second, validation of only three genes (ZC3H13, LRPPRC, IGFBP3) in 32 paired tissue samples is insufficient for comprehensive clinical validation, and our validation is restricted to mRNA levels rather than protein expression. Third, our study lacks mechanistic experiments to elucidate direct causal relationships between m6A modifications and observed phenotypes. Fourth, the discrepancy between modest AUC values (0.64–0.67) and high hazard ratios (8.59–14.27) suggests potential overfitting, particularly given the complex 59-gene model architecture, requiring cautious interpretation and independent validation. Additionally, extensive multiple testing across genes, immune populations, and clinical variables increases Type I error risk despite correction methods. Future studies should prioritize prospective cohort validation, functional mechanistic investigations, protein-level validation in larger cohorts, and integration of m6A signatures with existing clinical decision-making tools to enhance personalized treatment strategies for colorectal cancer patients.

## Supplementary Material

Supplementary Material

Supplementary Material

Supplementary Material

Supplementary Material

Supplementary Material
