# Supplementary figures and images for: Comprehensive analysis of m6A RNA methylation regulators for prognostic risk stratification and immune microenvironment characterization in colorectal cancer
Source: Open Med (Wars). 2026 May 7;21(1):20251318. doi: 10.1515/med-2025-1318 (PMC13150367; doi:10.1515/med-2025-1318)

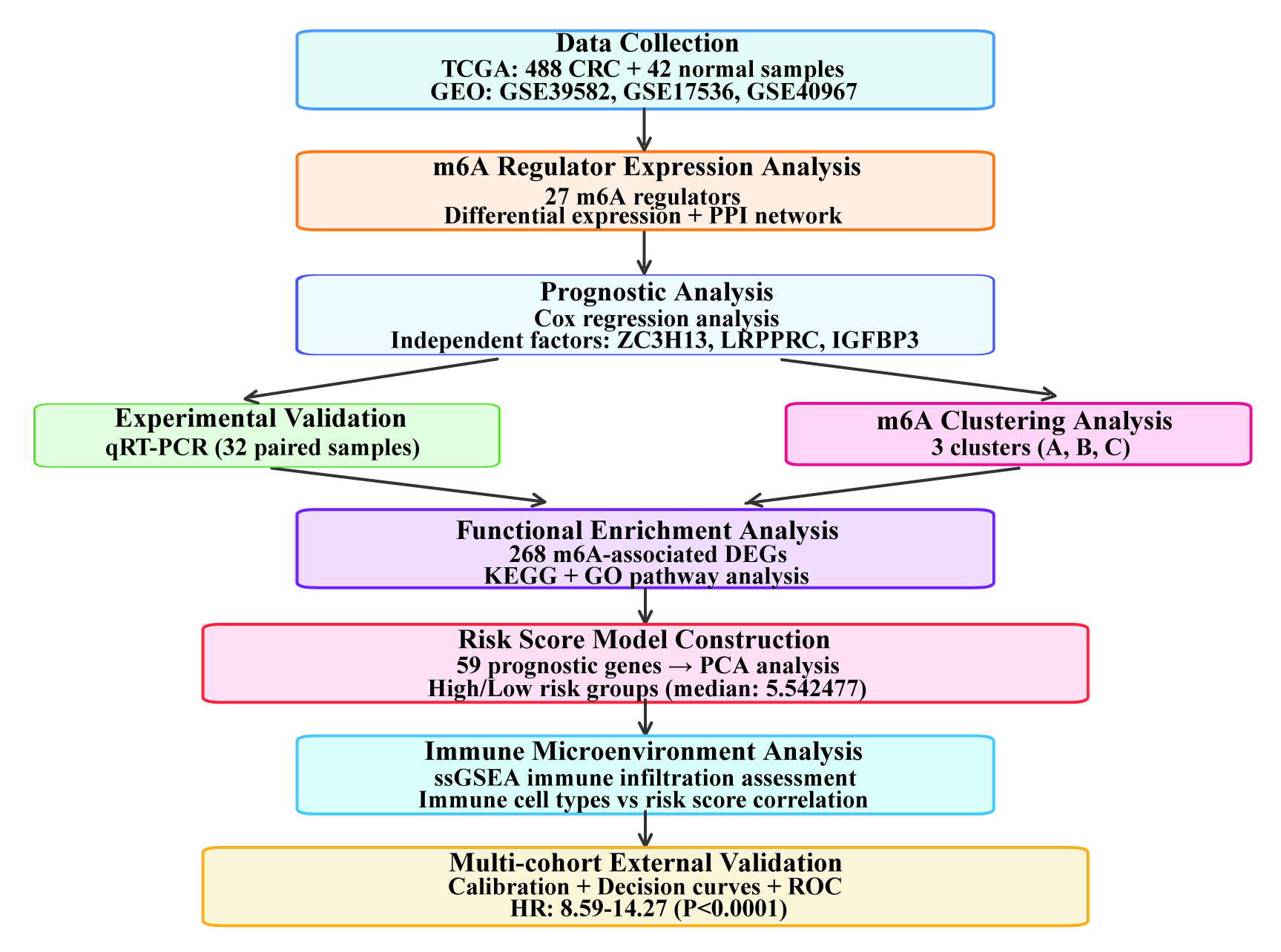

Supplement: Supplementary file 2 — Supplementary Material [file j_med-2025-1318_suppl_002.zip › j_med-2025-1318_suppl_002.tif]
